# Supplementary material for: Antibody gene features associated with binding and functional activity in vaccine-derived human mAbs targeting malaria parasites
Source: bioRxiv. 2023 Aug 3:2023.08.01.551554. Preprint. [Version 1] doi: 10.1101/2023.08.01.551554 (PMC10541113; doi:10.1101/2023.08.01.551554)
Supplement: Supplement 1 [file media-1.pdf]

SUPPLEMENTARY MATERIAL

Camila H. Coelho, Susanna Marquez, Anne D. Berhe, Bergeline C. Nguemwo Tentokam, Kazutoyo Miura, Carole A. Long, Sara Healy, Issaka Sagara, Steven H. Kleinstein, Patrick E. Duffy

Contents:

Supplementary Figures 1-4

Supplementary Table 1

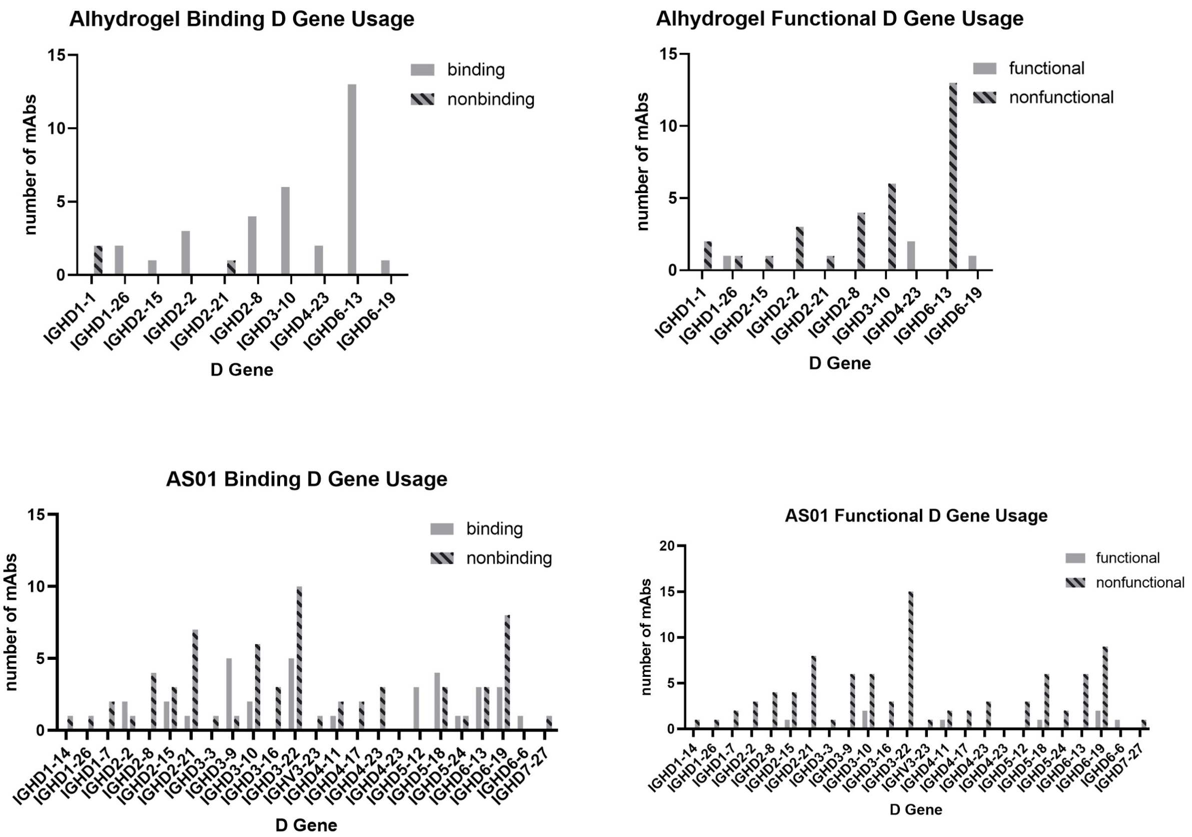

Supplementary Figure 1– D genes present in mAbs, grouped by binding and functional activity profile. Binding was assessed by ELISA and functional activity by SMFA. Functional antibodies are reported as with functional activity higher than 75% at 100µg/mL.

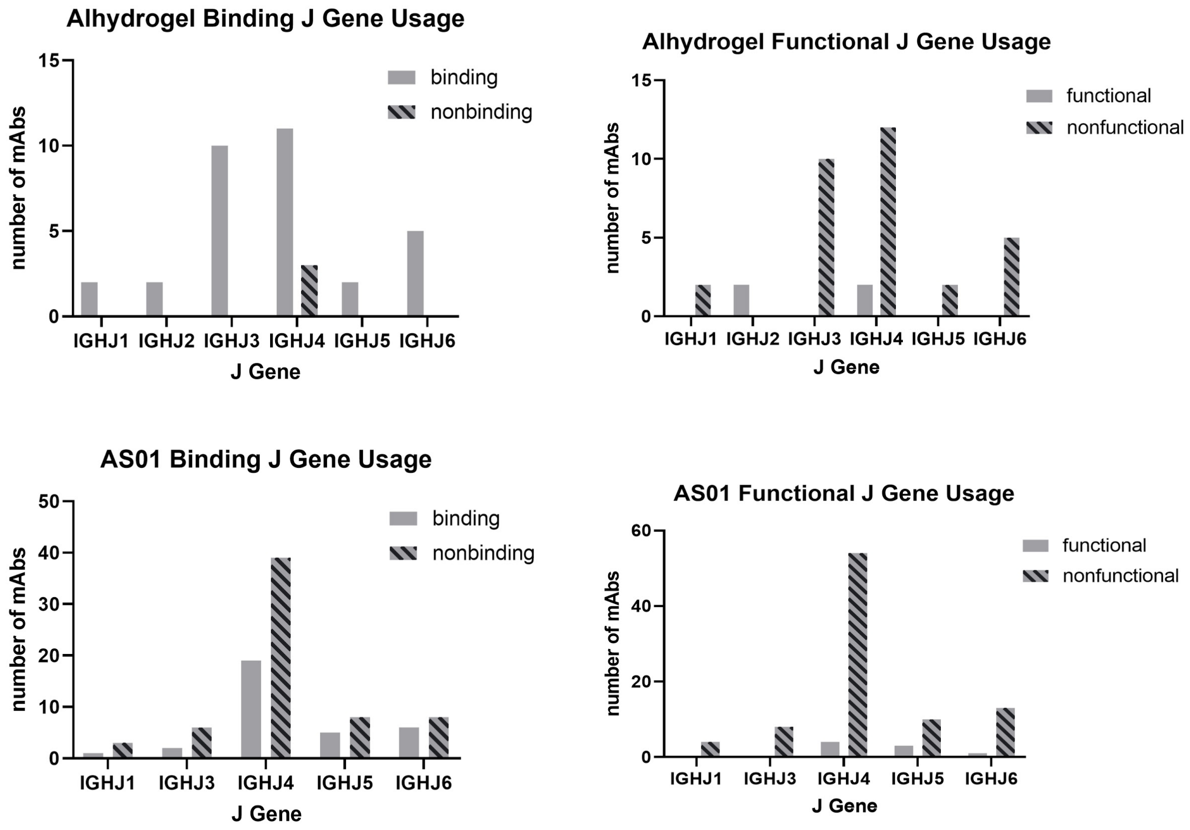

Supplementary Figure 2 – Heavy chain J genes present in mAbs, grouped by binding and functional activity profile. Binding was assessed by ELISA and functional activity by SMFA. Functional antibodies are reported as with functional activity higher than 75% at 100µg/mL.

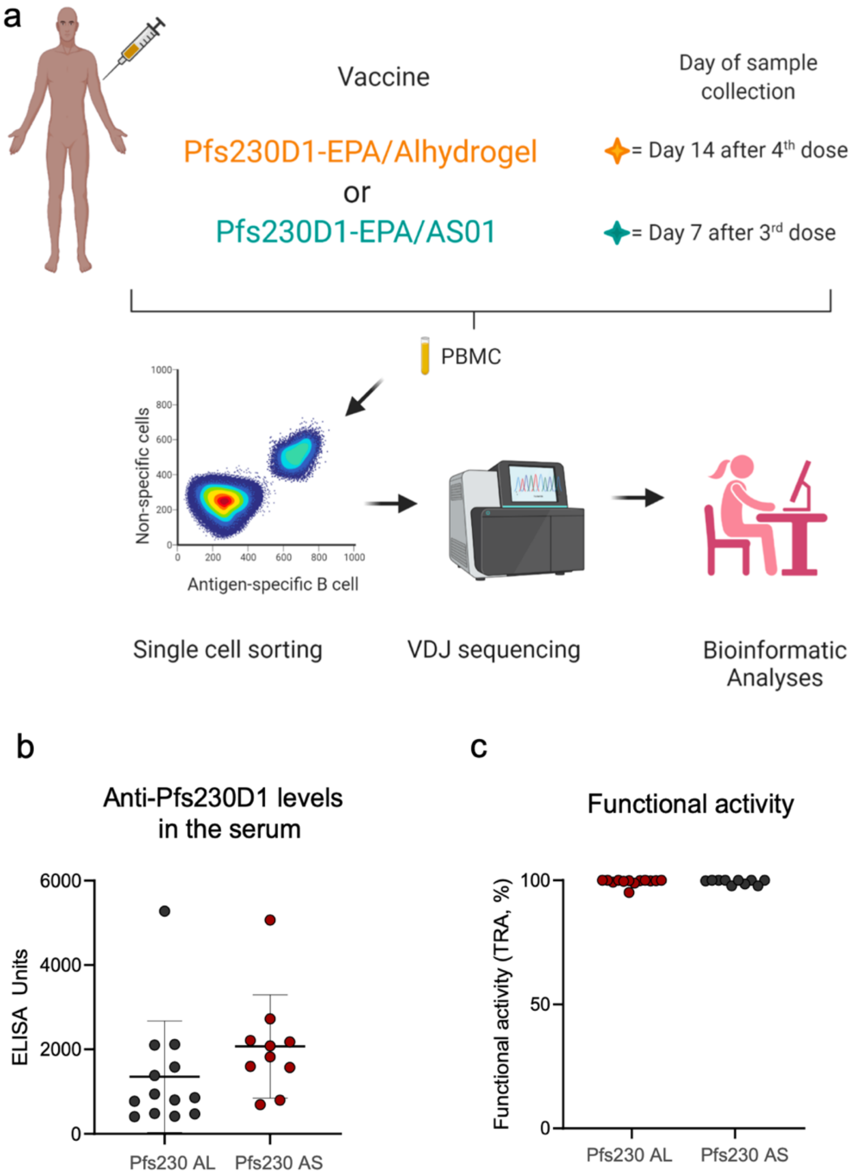

Supplementary Figure 3- Antibody repertoire in Pfs230D1-specific single B cells in response to vaccination with Alhydrogel or AS01 adjuvants. **(a)** Malian adults received 3 or 4 doses of Pfs230 conjugated with the carrier Exoprotein A and formulated with either Alhydrogel® or AS01 adjuvants. PMBCs were collected from subjects receiving Pfs230D1-EPA/Alhydrogel® (Pfs230AL) or Pfs230D1-EPA/AS01 (Pfs230 AS) and Pfs230D1-specific single B cells were sorted and had their B cell receptor sequenced. Bioinformatic analyses were performed using the Immcantation framework. Samples from the subjects enrolled in the clinical trial with vaccines formulated with Alhydrogel® were collected 14 days after the 4th dose, and with AS01, were obtained 7 days after the 3rd dose **(b)** Anti-Pfs230D1 IgG titers in response to both vaccines were measured by ELISA. **(c)** Serum functional activity was assessed by SMFA and determined by the ability to reduce the number of oocysts in midguts of infected *Anopheles* mosquitoes fed with *NF54 Plasmodium falciparum*.

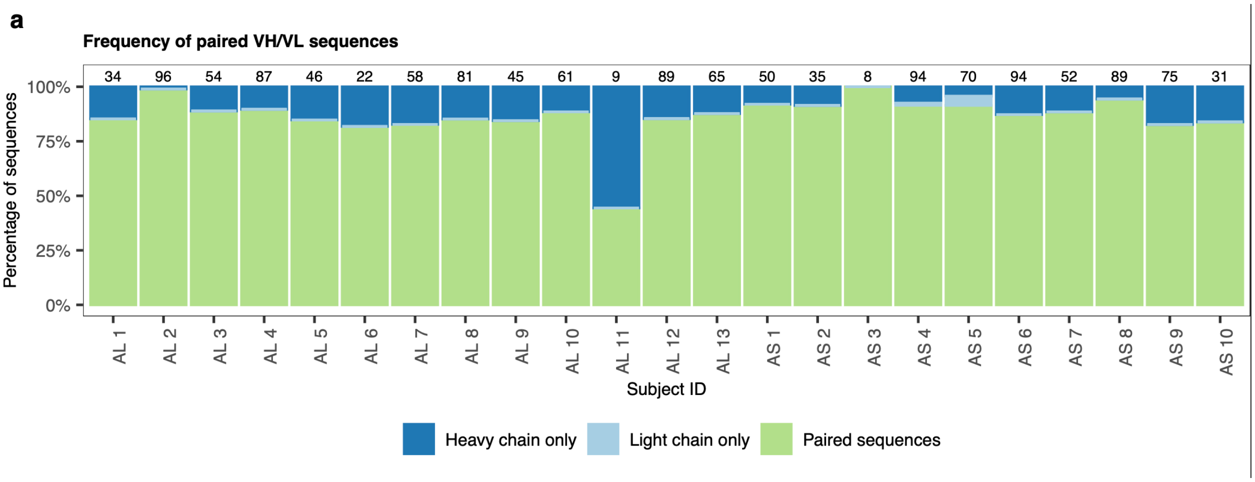

Supplementary Figure 4 - Proportion of Heavy chain (VH), light chain (VL) and pairs of VH/VL. Values in the Y axis represent the percentage for each of these three groups of sequences.

SUPPLEMENTARY MATERIAL (TABLES)

Alhydrogel (Subject ID)	Initial number sequences	Number of sequences after filtering	AS01 (Subject ID)	Initial number of sequences	Number of sequences after filtering
**1**	*69*	*34*	**1**	*69*	*50*
**2**	*183*	*96*	**2**	*55*	*35*
**3**	*112*	*54*	**3**	*16*	*8*
**4**	*148*	*87*	**4**	*152*	*94*
**5**	*98*	*46*	**5**	*123*	*70*
**6**	*71*	*22*	**6**	*119*	*94*
**7**	*106*	*58*	**7**	*78*	*52*
**8**	*154*	*81*	**8**	*165*	*89*
**9**	*82*	*45*	**9**	*109*	*75*
**10**	*125*	*61*	**10**	*52*	*31*
**11**	*37*	*9*			
**12**	*157*	*89*			
**13**	*117*	*65*			
TOTAL:	***1459***	***747***	TOTAL:	***938***	***598***

Supplementary Table 1 – Number of total BCR sequences per subject, including both VH and VL sequences.
